# Refining the COPES to Measure Social Climate in Therapeutic Residential Youth Care

**DOI:** 10.1007/s10566-017-9424-z

**Published:** 2017-11-21

**Authors:** Jonathan D. Leipoldt, Nanna S. Kayed, Annemiek T. Harder, Hans Grietens, Tormod Rimehaug

**Affiliations:** 1 Special Needs Education and Youth Care Unit, University of Groningen, Grote Kruisstraat 2/1, 9712 TS Groningen, The Netherlands; 2 Regional Centre for Child and Youth Mental Health and Child Welfare, Norwegian University of Science and Technology, PB 8905, MTFS, 7491 Trondheim, Norway; 3 Child and Adolescent Psychiatry Department, Nord-Trøndelag Hospital Trust, PB 333, 7601 Levanger, Norway

**Keywords:** Social climate, COPES, Therapeutic residential youth care, Questionnaire refinement, CFA, IRT

## Abstract

**Background:**

Previous studies have shown that social climate in therapeutic residential youth care (TRC) is important to the welfare of residents, staff, and assessing treatment outcomes. The most influential theory on social climate in residential settings is the theory of Moos. The measurement of the concepts and aspects of this theory using the Community Oriented Programs Environment Scale (COPES) has repeatedly been criticized regarding usability, validity, and reliability, especially for TRC.

**Objective:**

To improve the usability and psychometric quality of the COPES by shortening and refining the original subscale structure for usage in TRC.

**Methods:**

Four-hundred adolescents living in Norwegian TRC participated. We supplemented confirmatory factor analysis (CFA) with item response theory (IRT) to evaluate model fit, investigate factor loadings, and shorten scales to improve their psychometric qualities and usability in describing social climate in TRC.

**Results:**

The original subscales were not acceptable as evaluated by the criteria for CFA and IRT. By removing psychometrically weak items, the instrument was shortened to 40 items within the original ten subscales. This short version showed acceptable psychometric qualities based on both CFA and IRT criteria and the instrument retained its content validity. Finally, the original three higher-order dimensions was not supported.

**Conclusions:**

Compared to the original instrument, the refined 40-item version of the COPES represents a more usable instrument for measuring social climate in TRC. Future studies are needed to confirm the multifaceted refined short version in comparable samples of youth and staff to further investigate predictive value and construct validity.

## Introduction

Therapeutic residential youth care (TRC) concerns the treatment and care of young people outside their family environments. It aims to provide services to protect, care for, and prepare young people to return to life outside the institution (e.g., Harder and Knorth 2015). These young people have been unable to live at home due to problems on the part of parents, child abuse and neglect, or severe behavioral problems (Handwerk et al. 1998; Knorth et al. 2008; Whittaker et al. 2016). Treatment usually takes place within a therapeutic holding and learning environment (Hair 2005), and institutions are increasingly adhering to evidence-based treatment interventions (De Swart et al. 2012). We distinguish TRC from other types of residential care with other primary purposes, including detention, isolation, and basic care (e.g., prisons and orphanages). The defining characteristic is the inclusion of a pronounced “therapeutic” component.

Meta-analyses on outcomes in residential care (e.g. De Swart et al. 2012; Grietens 2002; Knorth et al. 2008; Scherrer 1994) have revealed small to moderate effects on improvement in emotional problems, a decrease in externalizing behavior problems, and less recidivism of delinquent behavior among adolescents in residential care. In contrast, long-term results indicate that, comparable to psychotherapy in general, the effects of interventions become less convincing as the length of the follow-up period increases, with short-term effects exhibiting more positive results (Frensch and Cameron 2002; Harder and Knorth 2015; Knekt et al. 2016; Scherrer 1994). Moreover, only limited evidence is available concerning how residential care actually achieves treatment goals: residential care remains too much of a “black box” (e.g., Harder and Knorth 2015; Knorth 2003; Knorth et al. 2008; Libby et al. 2005).

To increase the durability of positive treatment results, we need to know more about how results are achieved, rather than about the results that have been achieved (Harder and Knorth 2015). One of the factors associated with the process of behavioral change through treatment is the social environment within TRC institutions (hereafter denoted as social climate). Originating from the field of social ecology, the concept of social climate assumes that the behavioral regulation of an individual is not determined solely by personality characteristics and individual needs, but also by the demands of the near social environment (Feagans 1974; Murray 1938; Stern 1970). Social climate can therefore be defined as the discretion, consistency, and continuity of events that convey environmental influence on individuals in the shared environment.

Previous studies have illustrated the importance of a positive social climate in TRC by demonstrating its association with fewer social and behavioral problems in young people during care (Attar-Schwartz 2008), improved adolescent mental health (Timko et al. 2000), higher client satisfaction (Mesman Schultz 1992), more positive coping strategies, lower rates of peer victimization during care (Pinchover and Attar-Schwartz 2014), less runaway behavior (e.g., Attar-Schwartz 2013), lower criminal recidivism rates (Van der Helm 2011), and better working conditions for staff members (Glisson and Green 2006, 2011; Theunissen 1986; Williams and Glisson 2014). These associations suggest that social climate is important to the effectiveness of residential treatment programs (Andrews 2011; Cantora et al. 2014; Lanctôt et al. 2016).

Despite its importance, social climate has been conceptualized and specified in many different ways, drawing on both general and specific concepts. General conceptualizations of social climate include relational climate, psychosocial environment, social atmosphere, social environment, and ward atmosphere (Brunt and Rask 2012; Moos 1974b; Tonkin 2015). These conceptualizations have led to the development of a variety of specifications in scales and dimensions for measuring social climate. Most of these instruments have been developed in connection with specific treatment settings and client groups (Tonkin 2015). Social climate can also be measured by assessing the actual, preferred, and expected social climate and how it is experienced by residents, staff members, or external observers (Moos 2003). Social-climate assessment instruments specifically designed for TRC are currently limited (Theunissen 1986; Tonkin 2015). Most of the instruments that are available have been adapted from adult populations, and this weakens their predictive validity in TRC. The aim of the present study is therefore to assess and improve the psychometrics and usability of the most commonly used instrument in residential care: the Community Oriented Programs Environment Scale (COPES; Moos 2009). This instrument was selected for the present study in 2010 by a cross-disciplinary expert group in Norway, which had identified it as the best available instrument for measuring social climate in the context of a large research project on Norwegian TRC.

The COPES and its predecessor, the Ward Atmosphere Scale (WAS; Moos and Houts 1968), were developed across several decades in the USA (Moos 1974a, 1988, 1996, 2009). The COPES items were adapted from the WAS to reflect therapeutic programs, and cultural adaptations have been made to expand the instrument’s applicability (Moos 2009). The many varied subscales (see Table 1) represent specific aspects of the global concept of social climate. They allow specificity in comparative studies of social climate and enable the profiling of institutional environments, while making it possible to draw connections to specific individual determinants, detect specific effects of environmental risk factors, and allow for specificity in examining how social climate interacts with treatment outcomes or other factors (Moos 2003, 2009). In the present study, therefore, we retain a broad array of subscales rather than reducing the COPES to a single global measure of social climate.

**Table 1:** COPES dimensions and subscales descriptions (Reproduced with permission from Moos 2009)

Subscale	Description
Relationship dimension	
1. Involvement	How active and energetic members are in the program
2. Support	How much members help and support each other and how supportive the staff is toward members
3. Spontaneity	How much the programme encourages open expression of feelings by members and staff
Personal growth dimension	
4. Autonomy	How well-sufficient and independent members are in decision-making and how much they are encouraged to take leadership in the program
5. Practical orientation	The extent to which members learn social work skills and are prepared for discharge from the program
6. Personal problem orientation	The extent to which members seek to understand their feelings and personal problems
7. Anger and aggression	The extent to which members argue with other members and staff, become openly angry, display other aggressive behavior
System maintenance dimension	
8. Order and organization	How important order and organization are in the program
9. Program clarity	The extent to which members know what to expect in their day-to-day routine and the explicitness of program rules and procedures
10. Staff control	The extent to which staff use measures to keep members under necessary controls

The COPES was originally constructed based solely on theoretical assumptions and item content validity (Moos 1974b). The original samples of adult in-patients and elderly residential settings (Moos 2009) yielded acceptable levels of internal consistency (Cronbach’s alpha). In the construction of the COPES, however, no use was made of a factor analytic approach using either exploratory factor analysis (EFA) or confirmatory factor analysis (CFA)—methods that have since become more common ways of determining the validity of instruments (Brown 2015). The instrument has been repeatedly criticized for having poor psychometric properties, poor factor structure, and poor usability when applied in other samples and settings. Criticisms of the instrument’s psychometric qualities concern a variety of issues, including the inability to replicate the factor structure (e.g., Brunt and Rask 2012; Squier 1994), comprehension problems when applied with young people (Theunissen 1986), double negation when combining content and answers (Slot et al. 1980; Theunissen 1986), culturally outdated items (Røssberg and Friis 2003b), and the time required to respond to 100 items (Middelboe et al. 2001). In response to these critiques, Ballen et al. (2001) suggest that every study using the COPES should conduct a new factor analysis. In addition, Middelboe et al. (2001) recommend constructing a shortened version of the COPES, as such questionnaires should be brief, concise, and focused in order to balance reliability and the burden on participants (Bjorner et al. 2004; Stanton et al. 2002).

Techniques for shortening and refining instruments have generally relied on the methods of classical test theory (CTT), including reliability maximization (Clark and Watson 1995) and factor analysis (Brown 2015). The problem with reliability maximization is that alpha-reliability assumes tau-equivalent measures, which is often not the case (Brown 2015; Floyd and Widaman 1995). Redundant and overlapping items can survive these tests, resulting in low construct validity (Boyle 1991; Smith and Stanton 1999). Røssberg and Friis (2003b) suggest removing 23 items while retaining the original scale structure of the WAS. In their analysis, however, they changed the answering options to a four-point scale, thereby increasing the variance required to perform such a reliability maximization analysis on the WAS. Factor analysis, in which items load on a latent construct, does not require any tau-equivalent measure, and it provides better evaluations of construct and discriminant validity when designing instruments (Brown 2015).

Multiple studies have suggested alternative factor structures for the COPES based on exploratory factor analysis (EFA). In a study conducted in supported group home facilities in Sweden, Brunt and Rask (2012) found a six-factor solution of the COPES after removing 12 items and the Anger and Aggression subscale due to communality and content overlap between items and subscales. In a study performed in in-patient psychiatric facilities, Squier (1994) proposed a solution consisting of three higher-order factors, with two of the higher-order factors each containing four lower-order subscales. The use of EFA in refining scales can nevertheless threaten content validity, and it may narrow and change the available array of social climate aspects represented by subscales.

In this study, we adopted a confirmatory strategy rather than an exploratory strategy for the COPES in order to maintain the original structure of the instrument and to avoid refining it from the beginning. The main reason for not redesigning the entire instrument has to do with the theoretical power of the broad scales to allow the evaluation of social climate in the context of TRC (Moos 2003). Previous studies have identified the following as important elements of social climate: support (e.g., Attar-Schwartz 2013; Heynen et al. 2017; Pinchover and Attar-Schwartz 2014), autonomy (Barton et al. 2006; Barton and Mackin 2012), practical orientation (e.g., Eltink et al. 2015), expressiveness (Towberman 1992), involvement (Towberman 1992), clarity (e.g., Eltink et al. 2015), order (Langdon et al. 2004), and control or strictness (e.g., Attar-Schwartz 2013; Langdon et al. 2004; Pinchover and Attar-Schwartz 2014; Van der Helm 2011). Future social climate research within TRC is therefore likely to be more beneficial if a broad scale structure is used to measure these elements.

Another more innovative approach to instrument refinement is item response theory (IRT; Bjorner et al. 2004; Edelen and Reeve 2007; Kline 2005; Reeve and Fayers 2005), which assesses the relationship between a respondent’s response on an item and its corresponding level of the latent variable. The IRT approach can be used to select items that cover all levels of a latent concept and exclude redundant items (Stanton et al. 2002). The aim is to identify items that ensure coverage of the entire range of the scale (Reeve and Fayers 2005). The main advantage of IRT is that it does not require many items to produce a reliable instrument, as long as the items that are selected cover the full range of the latent variable under investigation.

The advantages of CFA and IRT can be combined to detect the best items with which to represent a scale (Brown 2015; Flora and Curran 2004; Kamata and Bauer 2008). Information from factor loadings and IRT parameters from a two-parameter logistic IRT model can allow the construction of scales with good item clustering and scale-scope differentiation, thereby resulting in instruments that are precise, broadly valid, and relatively brief (Brown 2015; Edelen and Reeve 2007). To the best of our knowledge, this approach has currently not been applied to the COPES, and Brunt and Rask (2012) recommend using IRT in future refinements of the COPES.

### The Present Study

The aim of the present study is to evaluate the psychometric properties of the COPES and to construct a shortened version while retaining the original scale structure. To the best of our knowledge, no earlier studies have combined CFA and IRT in the refinement of the COPES within the original scale structure. Furthermore, few studies involving the COPES have focused on adolescents in TRC institutions, while most studies have focused on adult samples in supported housing facilities and prisons. Additional insight into important social climate factors could help to remedy the “black box” character of TRC (Harder and Knorth 2015). It could also identity frequently overlooked ecological aspects in TRC that explain variance when assessing youth outcomes (Attar-Schwartz 2009).

We address the following research questions regarding the reliability, factor structure, and validity of the COPES: (1) How does the original COPES structure of subscales and dimensions function in a sample of adolescents living in TRC in Norway? (2) Is it possible to construct a refined, shortened version of the COPES with acceptable psychometric properties for measuring social climate in TRC using a combination of criteria for CFA and IRT?

For the first question, we expect the original COPES subscales and dimensions to produce highly variable and, in most cases, low-quality results when evaluated according to criteria for both CFA and IRT. With regard to the second question, we expect a shortened version of the COPES refined by combining CFA and IRT to produce a more usable instrument with improved psychometric qualities and retained content validity.

## Method

### Treatment Setting

In Norway, TRC institutions approved by the Directorate of Children and Family Affairs can be commercial, non-commercial, or publicly owned. These institutions are specialized in four areas of expertise: acute, care, conduct, and substance problems. Adolescents can be placed in TRC facilities due to abuse, neglect, or behavioral problems. Norwegian TRC institutions are small, typically hosting three to five adolescents per unit. Some are located in rural areas, while others are located in small towns or larger cities. The primary goal is to provide ordinary care and parenting substitution, with the goal of reducing the social and psychological problems of the residents, while helping to socialize them through relationships with staff members and the resident group. The basic policy is to provide daily routines that are as close as possible to those experienced in family care: attending school or work and participating in leisure activities both inside and outside the institution. The social climate of the institution is thus considered a core element of the treatment. The treatment may also include specific interventions for specific problems. Most staff members have degrees in social sciences, and they follow a milieu-therapeutic model in the institution. Individual psychiatric/psychological treatment is not provided by the institutions, but by mental health services in the community or in outpatient clinics organized by the Directorate of Health. Referrals are required for the assessment, diagnosis, and treatment of mental health problems (Jozefiak et al. 2015).

### Participants

**Fig. 1:**
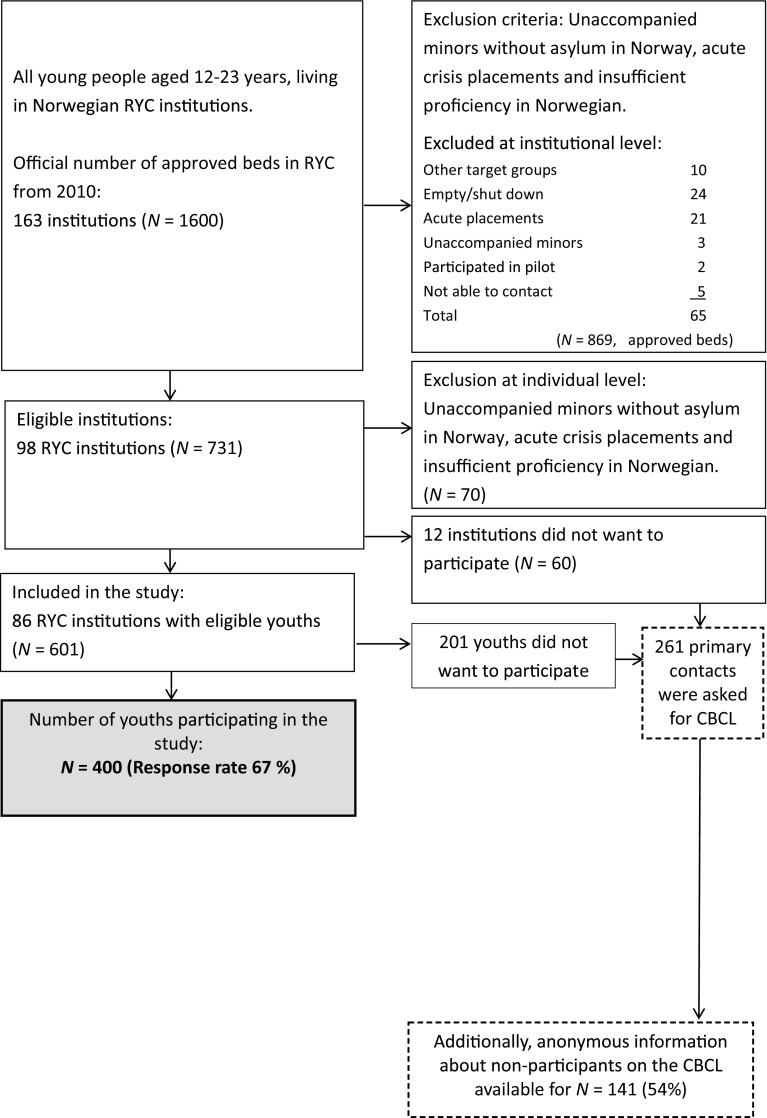
Inclusion flowchart for participants (Reproduced with permission from Jozefiak et al. 2015)

We obtained data for the present study from a large-scale Norwegian project on mental health in children and adolescents living in TRC (Jozefiak et al. 2015). All TRC units for adolescents aged 12–23 years were invited to participate in the study (see Fig. 1). Adolescents living in emergency care units and unaccompanied minors without asylum in Norway were excluded from participation, as they were considered to be in such a high state of crisis that data collection should not be a priority. We also excluded several institutions specialized in conduct problems (Andreassen 2015) because of their high level of internal research activity. In addition, adolescents with insufficient proficiency in Norwegian were excluded. Of the 98 eligible institutions and 601 eligible adolescents, 86 institutions and 400 adolescents consented to participate in the study, resulting in a response rate of 67% (Jozefiak and Kayed 2015).

Of the adolescents participating in the present study, 57.5% were female. The average age was 16.7 years (*SD* = 1.4), ranging from an average of 16.5 years (*SD* = 1.5) for males to 16.9 years (*SD* = 1.2) for females. Most of the adolescents attended school (69.2%) or had jobs (11.3%), and 76% met criteria for at least one psychiatric diagnosis. For further details about the sample characteristics, see Jozefiak et al. (2015).

### Measures

#### Social Climate

To measure social climate, we used a Norwegian version of the COPES that was designed to measure self-reported current experienced environment (Moos 2009). Although we also used a proxy version of the COPES for staff members, we do not address it in the present study. The COPES consists of 100 true/false statements about social climate in the institution. Taken together, the items form 10 subscales grouped under three dimensions. The subscales and dimensions are already described in the introduction (see Table 1). The following is an example item from the involvement scale: “The members are proud of this program.” An example item from the problem orientation scale is: “Members are taught specific new skills in this program.” The reliability and construct validity of the COPES have been well documented in adult clinical inpatient settings, with internal consistencies ranging from α = .58 for the staff control scale to α = .78 for the involvement scale (Moos 2009).

### Procedure

All Norwegian TRC institutions listed in the 2010 national database of the Directorate for Child and Family Affairs were contacted in random order. Institutions were provided with information about the research project and its goals. Data collection was carried out between 2011 and 2014 by four research assistants, all of whom held Master’s degrees in psychology or social work, and had extensive experience working with adolescents and families. They administered multiple questionnaires and interviews to adolescents, teachers, primary contacts (staff member), and institutional directors. Only self-reports are used in the present study.

The COPES was administered as the second instrument in the data-collection process, immediately after the initial interview, given its importance to the main study. Participants completed the COPES in the presence of the research assistant, and they were allowed to ask clarification questions regarding the content of the items. If the participants had difficulty reading the questions, the research assistant would read it aloud for them. After administering the COPES, the research assistants administered questionnaires concerning mental health, social support, and quality of life. Data collection lasted about 4 h for each participant and could be divided over 2 days. During the entire data-collection period, a team of child and adolescent psychiatrists and psychologists was available in case of emergencies. Each adolescent received a gift certificate of 500 Norwegian Crowns (approximately USD90) for participation.

### Ethics

Participants were recruited in accordance with procedures approved by the Norwegian Regional Committee for Medical and Health Research Ethics (reference number 2016/1169/REC Central). A standardized information and invitation letter was sent to the adolescents. In simple language, this letter described the information to be assessed, stressing that participation was completely voluntary and that consent could be retracted at any time. Written consent was always obtained from the adolescents. For adolescents younger than 16 years of age, informed consent was also obtained from a significant caregiver. At the start of the data-collection process, the details of the research project were discussed with the adolescent once more to ensure informed consent.

### Data Analysis

On average, missing items accounted for less than 5% of the values in all subscales, and only two participants responded to less than 66% of the items (there were no complete non-responders). Missing-value analysis based on Little’s test for missing data indicated that data was missing completely at random. We therefore decided to substitute missing values with the single-imputation approach of “estimation maximization” (Dempster et al. 1977). Items were substituted by subscale, and only information from that specific subscale was used to substitute missing data points.

We then conducted a confirmatory factor analysis (CFA) with the weighted least squares means and variances (WLSMV) estimation method for categorical indicators to evaluate the original structure of the COPES (Brown 2015; Muthén and Muthén 1998–2012). The variance–covariance matrix was estimated using tetrachoric correlations (Y*) (e.g., Brown 2015). Given our intent to respect the original structure, and given the sample-size issues associated with the WLSMV method (Flora and Curran 2004), all subscales were first assessed individually before testing the complete model. We used the first item of each factor as a marker variable to identify the model. To assess model fit, we used the Chi square test and Chi square/degrees of freedom (χ^2^/*df*) index, comparative fit index (CFI), Tucker–Lewis Index (TLI), and the root mean square error of approximation (RMSEA) with its 90% confidence interval. Based on the recommendations of Yu (2002) and of Hu and Bentler (1999), we considered a non-significant Chi square test, a (χ^2^/*df*) index < 2, CFI and TLI > .90, and a RMSEA of < .06 as indicators of good model fit. To determine the reliability of the subscales, we used Raykov’s reliability coefficient (RRC; Raykov 1997), with values > .70 indicating good reliability and values between .6 and .7 considered acceptable (Bagozzi and Yi 1988).

We performed the refinement through a four-step iterative process, which we conducted separately for each subscale. First, single factor models with all original items were used in a CFA to evaluate the initial model fit of these subscales. Second, we removed items that showed non-significant factor loadings or low standardized factor loadings. We considered standardized factor loadings of > .45 acceptable (e.g., Hair et al. 1995; Stanton et al. 2002) and omitted items with loadings < .45. Third, we investigated correlated errors for item pairs on content for modification indices with values of > .10. If the content showed overlap, we omitted the item of the correlated pair that least matched the original subscale description. Fourth, based on IRT methodology, items with low discriminatory ability (a < .65) were removed from the factor (Baker 2001). We also excluded overlapping items and outliers in item-difficulty parameters (< 2 or > 2). Items that least matched the content description for the original scale were removed when the process launched two alternative candidates for removal.

Following the aforementioned steps, we tested the model fit of two models that included only the remaining items. First, we estimated a measurement model that allowed covariation of the subscales. Second, we performed a second-order factor analysis using a model in which the three dimensions were regressed on their respective subscales.

Imputation of missing values was conducted in IBM SPSS version 23, and the CFA and IRT analyses were carried out in MPlus version 7.11 (Muthén and Muthén 1998–2012). For all analyses, alpha levels of < .05 were considered statistically significant.

## Results

The standardized factor loadings, item discrimination parameters, and item difficulty values for all the original subscales are displayed in Table 2. Because the COPES is protected by copyright, only significant keywords in the items are displayed. For full item content, the reader is referred to the manual for the COPES (Moos 2009).

**Table 2:** Factor loadings, IRT parameters and model-fit indices of the COPES subscales (Reproduction by special permission of the Publisher, Mind Garden, Inc., www.mindgarden.com from the Community Oriented Programs Environment Scale by Rudolf. Moos Copyright © 1974, 1988, 1996 by Rudolf H. Moos Further Reproduction is prohibited without the Publisher’s written consent)

Subscale and items	*λ*	a	b	*χ* ^2^ (*df*)	*p*	*χ* ^2^/*df*	CFI	TLI	RMSEA	90% CI RMSEA
1. Involvement				81.86 (35)	< .001	2.34	.95	.94	.058	.042–.074
**Energy**	.55	.66	− .63							
**Lively**	.74	1.09	− .79							
**Proud**	.82	1.41	.40							
Spirit^c^	.57	.67	− .40							
Youths volunteer^b^	.54	.64	.37							
Passing time^a^	.39	.42	.49							
Social activities^c^	.71	1.00	.08							
Busy^b^	.51	.60	.39							
**Interesting**	.59	.73	− .21							
Weekend activities^c^	.56	.67	− .18							
2. Support				248.11 (35)	< .001	7.09	.74	.67	.123	.109–.138
Take care^a^	.48	.55	− .93							
**Staff time**	.71	.99	− .58							
Youths help^a^	.41	.45	− 1.59							
**Follow-up**	.57	.69	− .87							
**Staff compliments**	.76	1.19	− .91							
Youths want^c^	.64	.83	− .16							
Staff show-up^a^	.49	.55	− .63							
Youths sharing^a^	.35	.37	− .91							
**Individual attention**	.70	.99	− .47							
Youths acquainted^b^	.63	.81	− 1.09							
3. Spontaneity				105.87 (35)	< .001	3.02	.83	.78	.071	.056–.087
Hide feelings^c^	.48	.55	.10							
**Say anything**	.52	.61	− 1.87							
Youths feel^c^	.54	.63	.15							
Youths careful^4^	.51	.60	.20							
**Free expression**	.50	.57	− 1.03							
Spontaneous activities^a^	.36	.39	− 2.94							
Hide disagreements^a^	.31	.33	− 1.55							
Like doing^a^	.41	.44	− .33							
**Hide feelings**	.76	.16	.33							
**Express feelings**	.53	.62	− .35							
4. Autonomy				75.92 (35)	< .001	2.17	.85	.81	.054	.037–.071
**Youth government**	.69	.95	− 1.19							
**Influence rules**	.76	1.15	.37							
Expected leadership^a^	.41	.45	− 3.29							
Discourages criticism^a^	− .09	− .09	− 3.76							
Free leave^a^	.23	.24	3.55							
Take charge^a^	.35	.37	− 1.13							
**Suggestions**	.62	.79	− .10							
**Responsibilities**	.45	.51	− .26							
Independent^c^	.46	.51	− .22							
Staff ideas^a^	.19	.20	.62							
5. Practical orientation				162.82 (35)	< .001	4.65	.74	.67	.096	.081–.111
Training jobs^b^	.51	.60	− 1.07							
**Practical problems**	.46	.52	− .95							
Future plans^a^	.41	.45	− .22							
**Follow-up discussions**	.63	.82	.22							
**Follow-up plans**	.66	.89	.04							
Focus past^a^	.17	.17	− .95							
Demonstrate progress^a^	.39	.42	− 1.75							
**Taught skills**	.72	1.05	− .38							
Encourage community^b^	.45	.50	− 1.11							
Plan leaving^a^	.44	.50	.34							
6. Personal problem orientation				138.99 (35)	< .001	3.97	.77	.71	.086	.071–.101
Sex talks^a^	.34	.36	.33							
Problem talks^a,f^	.64	.84	.07							
**Personal questions**	.51	.60	− .60							
**Share problems**	.48	.55	1.73							
Talk past^c^	.47	.52	− .11							
Express anger^4^	.60	.75	.17							
Focus feelings^a^	.35	.38	− 2.01							
Personal problems^f^	.59	.73	− .25							
**Discuss problems**	.44	.48	.35							
**Past talks**	.54	.63	.84							
7. Anger and Aggression				64.26 (35)	.002	1.84	.94	.92	.046	.027–.063
Argue^a^	.24	.25	− 2.61							
Critize joke^a^	− .43	− .47	1.25							
**Arguments**	− .69	− .96	− 3.01							
Staff argues^a^	− .37	− .40	− 3.44							
Practical jokes^a^	− .17	− .17	10.30							
Openly angry^a^	.16	.16	3.44							
**Staff starts**	− .56	− .68	.55							
**Youths gripe**	− .73	− 1.07	.19							
Argue healthy^a^	− .11	− .11	− 9.20							
**Angry**	− .92	− 2.29	− .61							
8. Order and organization				79.68 (35)	< .001	2.28	.94	.92	.056	.040–.073
Activities planned^b^	.50	.58	− .21							
Organized program^e^	.67	.90	− .46							
**House neat**	.59	.73	− 1.22							
**House messy**	.67	.91	− .69							
Regular schedule^a^	.39	.43	− .37							
Youths messy^b^	.52	.61	.04							
**Disorganized**	.70	.97	.43							
**Dayroom untidy**	.78	1.24	− 1.05							
Appointments^a^	.44	.50	− .13							
Neat orderly^b^	.53	.62	− 2.31							
9. Program clarity				64.22 (35)	.002	1.84	.95	.94	.046	.027–.063
Rule consequences^a^	.43	.48	− 1.76							
**Changes explained**	.70	.97	− .42							
Detailed program^c^	.52	.62	− .25							
**Rules understood**	.54	.63	− 1.18							
Unpredictable^c^	.63	.81	.47							
**Staff presence**	.62	.80	− .74							
Charge^a^	.31	.32	− .40							
**Changing minds**	.65	.85	.39							
Ready leave^c^	.57	.69	.46							
Rule changes^c^	.62	.79	− .36							
10. Staff control				139.76 (35)	< .001	3.99	.65	.54	.087	.072–.102
**Follow schedule**	.61	.77	− 1.46							
Privileges punishment^a^	.27	.30	.09							
**Rule punishment**	.59	.73	− 1.28							
Fighting unacceptable^a^	.27	.23	− 4.20							
Youths ordered^a^	.15	.15	.91							
**Know rules**	.66	.87	− .72							
Free wear^e^	.56	.67	1.55							
**Interrupt staff**	.51	.59	− .71							
Discharge rules^a^	.13	.14	2.99							
Refuse activities^a^	.51	.60	1.59							

Items in bold are retained in final version. Items only show important keywords due to copyright

^a^Removed due to a non-significant or low factor loading

^b^Removed due to low discriminatory ability

^c^Removed due to overlapping item-difficulty parameters or non-significant values

^d^Removed due to a high modification indices

^e^Removed for other reason

^f^For the personal problem orientation scale there was indication that higher factor loadings became low after omitting the lowest loadings

### Scale-Reduction Process

Reasons for removing items are displayed in Table 2. Most items (36) were omitted due to a non-significant or low factor loading. Another 12 items were removed due to overlapping or non-significant difficulty parameters, and 8 items were removed due to low discriminatory ability. Two items were removed due to high modification indices, and one item was removed due to outdated content (“Wear whatever”). Finally, one item was removed due to non-convergence of the model, as illustrated in the following section.

### The Refined Short Version of the COPES

In all, we removed 60 items from the questionnaire, leaving 40 items, with four items for each factor. To test the quality of this refined short version of the instrument, we specified a measurement model that allowed covariation for all subscales. Initially, the model did not converge, due to a linear dependency in the matrix. We discovered that one item (“organized program”) was highly correlated with other items and contained summarized content for the items with which it was highly correlated. We therefore replaced this item with the item “house messy,” which did not have the aforementioned content issues. It had comparable psychometric properties, but a pattern of lower covariance with other items. The model converged after the item was replaced, and the standardized factor loadings, model-fit indices, and composite reliability scores of the refined COPES are displayed in Table 3.

**Table 3:** Standardized factor loadings of the final COPES short version (Reproduction by special permission of the Publisher, Mind Garden, Inc., www.mindgarden.com from the Community Oriented Programs Environment Scale by Rudolf. Moos Copyright © 1974, 1988, 1996 by Rudolf H. Moos Further Reproduction is prohibited without the Publisher’s written consent)

Items	I	S	SP	A	PO	PPO	AA	OO	PC	SC
Proud	.933									
Lively	.741									
Interesting	.563									
Energy	.523									
Staff compliments		.823								
Staff time		.799								
Individual attention		.680								
Follow-up		.537								
Express feelings			.738							
Free expression			.616							
Hide feelings			.552							
Say anything			.360							
Suggestions				.741						
Youth government				.667						
Influence rules				.647						
Responsibilities				.477						
Taught skills					.858					
Practical problems					.634					
Follow-up plans					.571					
Follow-up discussions					.533					
Discuss problems						.751				
Past talks						.578				
Personal questions						.533				
Share problems						.438				
Angry							.799			
Youths gripe							.778			
Staff starts							.712			
Arguments							.643			
Disorganized								.824		
House neat								.681		
Dayroom untidy								.645		
House messy								.640		
Changes explained									.710	
Changing minds									.624	
Staff presence									.596	
Rules understood									.556	
Know rules										.883
Follow schedule										.778
Rule punishment										.354
Interrupt staff										.338
Total explained variance^a^	.50	.52	.34	.41	.44	.34	.54	.49	.39	.41
RRC value	.79	.81	.66	.73	.75	.67	.82	.79	.72	.70

All factor loadings are significant (*p* < .001)

Model-fit indices: *χ* ^2^ (*df*) = 951.71 (695), *p* < .001, *χ* ^2^/*df* = 1.37, GFI = .94, TLI = .93, RMSEA = .030 (90% CI .025–.035)

*I* involvement, *S* support, *SP* spontaneity, *A* autonomy, *PO* practical orientation, *PPO* personal problem orientation, *AA* anger, *OO* order and organization, *PC* program clarity, *SC* staff control, *RRC* Raykov’s reliability coefficient

^a^Proportion explained variance in y* explained by the factor. Average *R* ^2^ = .44

The Chi square test tends to become significant with a larger sample size. The other fit indices, which are less sensitive to sample size, provide a better indication of model fit than the Chi square test does (Hu and Bentler 1999). The model-fit indices demonstrate that the model fitted well (see Table 3). Composite reliability values suggest that the subscales can be measured reliably with four items as indicators. On average, the data explain 44% of the variance in the model, and the varying factor loadings are within range of acceptable values. All single items that have been criticized in previous publications for various reasons were excluded by our criteria, and the remaining items are well in line with the original scale-content description, as shown in Table 1. The highly criticized “anger and aggression” scale was retained with acceptable psychometric quality, but shows negative or non-significant correlations with other subscales.

The estimated correlations between the subscales in the refined short version are shown in Table 4. Five correlations are not significant, and the “staff control” subscale exhibits the lowest correlations with the other nine subscales.

**Table 4:** Correlations between estimated COPES subscales

Subscale	I	S	SP	A	PO	PPO	AA	OO	PC	SC
I	–	–	–	–	–	–	–	–	–	–
S	.83	–	–	–	–	–	–	–	–	–
SP	.65	.82	–	–	–	–	–	–	–	–
A	.86	.85	.76	–	–	–	–	–	–	–
PO	.62	.75	.62	.77	–	–	–	–	–	–
PPO	.35	.35	.71	.47	.45	–	–	–	–	–
AA	− .50	− .45	− .45	− .47	− .35	− .10^a^	–	–	–	–
OO	.54	.61	.48	.63	.57	.07^a^	− .41	–	–	–
PC	.78	.85	.69	.87	.71	.16^a^	− .70	.73	–	–
SC	.30	.25	.10^a^	.35	.24	.18	− .12^a^	.34	.28	–

All other correlations *p* < .05

*I* involvement, *S* support, *SP* spontaneity, *A* autonomy, *PO* practical orientation, *PPO* personal problem orientation, *AA* anger and aggression, *OO* order and organization, *PC* program clarity, *SC* staff control

^a^ *p* = ns

### Assessment of the Dimensions

Correlations between the subscales indicate that subscales belonging to the same dimension do not have systematically higher correlations with each other than they do with any other subscale (see Table 4). This indicates that a dimensional model would not necessarily be better than the first-order model. To test this assumption, we performed a higher-order factor analysis with three second-order factors (the three dimensions, as displayed in Table 1) loading on their respective subscales. Model-fit indices indicate that the dimensional model also fits well: χ^2^(*df*) = 1038.88 (727), *p* < .001, χ^2^/*df* = 1.43, CFI = .93, TLI = .92, RMSEA = .033, 90% CI (.028–.037). The standardized factor loadings for the dimension show high values (relationship-personal growth, *λ* = .99; relationship-system maintenance, *λ* = .87; and personal growth-system maintenance, *λ* = .94), thus indicating that the dimensions can be considered a single factor. Moreover, a difference test on the model fit indicates that specification of dimensions significantly worsens the first-order model: *χ* ^*2*^(*df*) = 89.87 (32), *p* < .001.

## Discussion

The aim of this study was to evaluate and shorten the COPES while retaining the original scale structure. The refinement was intended to increase the psychometric qualities of the instrument, retain validity, and increase its usability among adolescents in TRC. Drawing on data from a larger study conducted with 400 adolescents living in TRC facilities in Norway, we combined CFA and IRT methods to evaluate and refine the original scale structure of the COPES (Moos 2003, 2009). As expected, the original structure did not fit well, based on the CFA and IRT criteria for factor loadings, model-fit and parameters for item difficulty and item discrimination in this sample of adolescents living in TRC. Our refinement strategies succeeded in reducing the 10 scales into 10 shorter, well-functioning scales, each consisting of four items. All scales showed acceptable factor loadings, IRT parameters and model-fit characteristics and proved usable as a 10-scale model for measuring social climate.

### The Original Structure and Suggested Revisions

Our first hypothesis is confirmed by the finding that, for 6 of the 10 subscales, items matched the proposed scale concepts poorly, based on model-fit indices. In the other four subscales, for which the model-fit indices were acceptable, factor loadings indicated that some items in the scale did not perform well. In addition, the overlapping item-difficulty values within each scale reveal that many items measure the constructs at similar levels, thus indicating that the scope of each scale is represented in an overly homogeneous manner. Finally, several adolescents complained about the limited relevance of some items, indicating that some content is not well adapted to the context of TRC. None of the original scales meets all four criteria.

Our findings are consistent with those of previous studies, which have been unable to replicate the original structure with exploratory factor analysis (Brunt and Rask 2012; Middelboe et al. 2001; Røssberg and Friis 2003b; Slot et al. 1980; Squier 1994; Theunissen 1986; Van der Ploeg 1984). Some of these studies have proposed alternative factor structures to provide a suitable model for measuring social climate in supported group homes for young adults (Brunt and Rask 2012), for child protection facilities (Slot et al. 1980), and for in-patient psychiatric facilities (Squier 1994). In our sample, we were also unable to replicate the proposed new model based on data from supported group homes (Brunt and Rask 2012). This finding suggests that the context factors for social climate in TRC may differ from those in supported group homes. Group climate tends to focus more on smaller settings, which place higher priority on cohesion, repression, independence, task orientation, and control, while social climate is more concerned with broader perspectives, including the expression of feelings, involvement, autonomy, and problem orientation (Moos 2002; Strijbosch et al. 2014). Our inability to confirm previously suggested alternative structures supports our expectation that the original scale structure could be retained following refinement of the scales.

### The Refined Short Version of the COPES

Our second hypothesis was also confirmed, as we were able to increase the usability of the instrument by reducing the number of items by 60%. In addition, we improved the model fit with fewer items while retaining the subscale structure, scale scope, and range. We did this with far fewer items selected through a combination of CFA and IRT strategies, supplemented with content evaluations. Items disqualified by adolescents were excluded based on the other criteria. In our evaluation, the remaining items provide a good reflection of the original scale-content descriptions published by Moos (2003). The model-fit indices and composite reliability scores indicate that our proposed shorter version with 10 scales and 40 items has acceptable levels of construct validity, reliability, and scope for use in the context of TRC.

### Refinement of Especially Problematic COPES Subscales

Two of the subscales—“anger and aggression” and “staff control”—require additional attention. Brunt and Rask (2012) removed the “anger and aggression” subscale, because they found its content inconsistent and considered it irrelevant for supported group homes in a Swedish context. Røssberg and Friis (2003a) showed that this subscale is multidimensional, thus raising practical problems when using it as a measure of a single dimensional construct. They recommend creating a new scale (“angry and aggressive behaviors”). Furthermore, the interpretation of the core content of the scale is open to debate. Some items could mean that the institutions tolerate or do not suppress aggression, although they could also imply the presence of an unsafe and unprotected climate in which anger and aggression can be expressed with few consequences (Van der Helm 2011). In our refined version, we consider the core content to provide a clearer reflection of the extent to which negative aggressive behaviors contribute to the total score, which is well within the original scale description by Moos (2003). Due to the variety of behavioral and emotional problems experienced by young people living in TRC (Jozefiak et al. 2015), we are convinced that “anger and aggression” remains an important construct in assessing social climate. The refined “anger and aggression” scale is less capable of representing how staff members act on these behaviors and whether aggressive behaviors are repressed or tolerated (Røssberg and Friis 2003b; Van der Helm 2011). These aspects of social climate are partly addressed by the “staff control” and “order and organization” scales.

Consistent with previous studies, we found weaker correlations between the “staff control” subscale and the other subscales than we did for any of the other subscales (Brunt and Rask 2012; Røssberg and Friis 2003b). This raises the question of whether the “staff control” subscale belongs to the overall concept of positive social climate, although the model-fit indices suggest that it does. It also raises the question of whether any higher-order latent dimensions might cluster the COPES scales in ways other than those originally suggested by Moos (2003).

### Higher-Order Dimensions

The results provide no statistical support for using the original higher-order dimensions in our sample. Although we found acceptable model-fit characteristics when allowing the dimensions specified by Moos (2003) to load on their respective scales, allowing dimensions significantly worsened the model fit when compared to a single-level measurement model. A model with one higher-order social climate factor was also not supported by CFA criteria. Our final refined version of the COPES therefore consists of only 10 subscales, each consisting of four items, with no suggestions for calculate higher-order dimensions or a total score for positive social climate.

Previous studies have generated mixed evidence regarding the use of the dimensions. Theunissen (1986) was unable to detect the original higher-order dimensions in a factor analysis. Brunt and Rask (2012) reported that the dimensions could be used, but only based on acceptable internal consistency measures (Cronbach’s alpha), which do not test construct validity as CFA would. Based on principal component analysis, Piper, Rosie, Joyce, and Azim (1996) suggest a two-factor solution: (1) positive social climate and (2) perceived intensity and control of the expression of anger and aggression, drawing on data from an intensive psychodynamic group setting with adult residents. Van der Helm et al. (2011) make a similar suggestion for adolescents living in therapeutic youth prisons.

Our results indicate that the intercorrelations between the dimensions are so high that the dimensions cannot be treated as representing separate constructs. There is thus no statistical support for the contention that social climate of TRC institutions can be summarized using the three original dimensions or a single common score. In line with our statistical findings, Moos (2009) does not describe any calculation of dimension scores or suggest any summary score, having used the dimensions only as descriptive categorizations of the subscales. The results of our study demonstrate that the multifaceted evaluation of social climate according to 10 subscales has better statistical support than does any higher-order summary. A multifaceted approach could be better suited to exploring the relative importance of social-climate elements and resident characteristics in different TRC settings, as well as their predictive validity in relation to various treatment outcome factors (Piper et al. 1996). Rather than searching for general higher-order factors, future research should attempt to disentangle and deepen the specific understanding of many aspects of social climate and to search for clusters of social-climate aspects that have predictive value for specific outcomes.

### Strengths and Limitations

The primary strength of our study is the low rate of missing values and the relatively large sample size of 400 adolescents, which comes close to representing a population study of TRC residents in Norway and constitutes a large sample size for clinical studies. These features support the generalization of our results for use in the context of TRC, although the conclusions may have more limited value in other target groups or other institutional care and treatment arrangements. Compared to other social-climate instruments (Tonkin 2015), our refined version of the COPES allows for the specific assessment of highly diverse aspects of social climate in an institutional environment (Moos 2003). It could also entail an important improvement, as many instruments are currently lacking in psychometric quality and represent only a limited definition of social climate (Tonkin 2015). Another strength of the present study is the combination of methodologies used in the refinement process. Using CFA with the WLSMV estimator and IRT, we were able to compare item statistics for both factor loadings and IRT parameters to determine whether the items would constitute a good representation of a single shared concept, while reflecting a broad spectrum of that concept. An instrument shortened from 100 to 40 items could also reduce the burden on participants when the instrument is used, while enhancing its usability in assessment procedures and research (Bjorner et al. 2004; Stanton et al. 2002).

The main limitation of the present study is that the sample size necessitated testing the subscales separately when evaluating the original scales, as well as in the refinement process. Testing all scales and all remaining items in a model that allows covariation for all subscales was possible only after the refinement process. Based on the recommended sample size ([n * n + 1]/2; Brown 2015), the simultaneous analysis of all 100 items should ideally require 5050 participants, which far exceeds the population of adolescents in Norwegian TRC (SSB 2014). Given our intent to respect the original scale structure, we are convinced that it is fair to conduct the final CFA using only the remaining items. Nevertheless, the fit statistics for the original set of items might have been different if we had estimated a measurement model with all 100 items, allowing for second-order dimensions or a higher-order common factor. Future studies should therefore try to replicate our results in other populations to evaluate whether the refined model is usable, reliable, and valid in other settings, countries, and subpopulations.

A second limitation is that the items remaining in the COPES are subject to an imbalance resulting from positively formulated statements, even though we used statistical methods that are less biased (Stanton et al. 2002). Spontaneous comments from the participating adolescents indicated that they had more difficulty interpreting statements that were formulated negatively than they did with positive statements. This might explain why more negative items were omitted based on their psychometric performance. This might have generated a positively skewed portrayal of the social climate in the institutions (Streiner and Norman 2003). However, we preferred not to change the items. Despite its limitations, the present study demonstrates that a short, 40-item version of the COPES can be used as a reliable measure of social climate in the context of TRC.

### Implications for Research

Our study contributes to future research efforts by developing a better instrument for measuring social climate among adolescents in TRC institutions. In accordance with Moos (1990, 2009), we do not recommend using any single composite or dimensional score. The use of composite scores could disturb meaningful relationships to determinants of these aspects of social climate. This also applies to the specificity of predictive value related to outcomes of institutional care in interaction with client characteristics, care, and treatment aims. A multifaceted concept also makes it possible for new studies to specify which aspects of social climate are important for each research question. Future studies should investigate whether the use of more positively formulated questions produces a biased view of social climate. Furthermore, future studies should evaluate how staff members and leaders experience social climate, and whether the refined model can be used also to describe their scores. This would contribute to a more robust literature base concerning the validity of the COPES. Finally, the predictive value of the refined model should also be investigated with regard to associations with gender, age, type of placement, and mental health disorder. The present study was not intended to evaluate such associations and differences. Future studies could use multi-group CFA and differential item functioning to detect potential differences in the model. This could then be reflected in the construction of different norm groups.

### Practical Implications

Based on the psychometric properties and retained items in the COPES, the results of our study indicate that social climate can be described using a multifaceted approach. Such descriptions could be used to profile institutions in the manner suggested for the original version of the COPES. Our shortened version could also be used as a quicker, higher-quality way to obtain repeated specified measures of social climate over time, in order to identify environmental problems, evaluate institutional processes, and generate repeated feedback from residents with regard to how they perceive the social climate. Institutions should be profiled in terms of social climate using multiple scales rather than dimensional composites and composite scores that are not psychometrically sound. Such profiles could provide a better understanding of how different institutions promote development in adolescents, and could result in a better approach to comparing TRC environments and testing their associations with outcome variables.

## Conclusion

We are convinced that the refinement process that resulted in our 40-item version of the COPES has removed redundant, ambiguous, and less relevant items from the COPES, while improving its construct validity and reliability for research and assessment procedures evaluating social climate for young people living in TRC. We expect that insights from this study will contribute to discussions concerning the “black box” character of TRC by generating insight into “how things work in TRC” (Harder and Knorth 2015). Social climate is one of several ecological factors in the list of institutional correlates that are often overlooked and that could explain considerable variance in treatment outcomes (Attar-Schwartz 2009, 2017). A clearer picture of important social climate aspects could advance the existing knowledge on “what works for whom” in TRC, emphasizing the importance of examining institutional characteristics.
